# Barriers to Testing and Treatment for Chagas Disease among Latino Immigrants in Georgia

**DOI:** 10.1155/2012/295034

**Published:** 2012-12-30

**Authors:** Rebecca M. Minneman, Monique M. Hennink, Andrea Nicholls, Sahar S. Salek, Francisco S. Palomeque, Amina Khawja, Lauren C. Albor, Chester C. Pennock, Juan S. Leon

**Affiliations:** ^1^Rollins School of Public Health, Emory University, Atlanta, GA 30322, USA; ^2^Emory College, Emory University, Atlanta, GA 30322, USA

## Abstract

*Background*. The lack of testing and treatment of Chagas disease (CD), caused by Trypanosoma cruzi, amongst infected immigrants in the USA increases the risk of serious health complications and transmission (congenital or via blood transfusions). *Goal*. Our goal was to identify the barriers to testing and treatment of CD and understand the process of seeking healthcare amongst Latino immigrants in Georgia. *Methods*. In this qualitative study, eleven focus group discussions were conducted with 82 Latino immigrants, including migrant farm workers. Grounded theory was used to collect and analyze the data to develop an inductive conceptual framework to explain the context and process of seeking healthcare for CD amongst this at-risk population. *Results*. Participants were not aware of CD. Three healthcare seeking behaviors were identified: delaying treatment, using traditional remedies, and using either mainstream or alternative health providers. Behaviors and motivations differed by gender, and the use of licensed medical providers was considered a last resort due to the cost of healthcare, loss of earnings while seeking care, and fear of diagnosis with fatal illness. *Discussion*. Providing free or low cost services, mobile clinics, and education regarding CD is critical to increase testing and treatment of CD in the US.

## 1. Introduction

In the United States, the prevalence and burden of Chagas disease are increasing [1]. In the USA, the population most at-risk for developing Chagas disease, caused by the parasite *Trypanosoma cruzi*, are Latin American immigrants who became infected with *T. cruzi* in their home countries through vector borne transmission from triatomines, infants born of *T. cruzi* infected mothers (i.e., congenital transmission), and, rarely, individuals infected through autochthonous transmission (reviewed in [2–4]). The USA is the country with the highest number of Latin American immigrants [5]. In various immigrant populations, including migrant farm workers, seroprevalence rates ranging from 0.4% to 4.9% have been documented [6–8]. Further, it is estimated that approximately 300,167 Latin American immigrants in the USA are infected with *T. cruzi* [9]. If left untreated, *T. cruzi* infection may result in a dilated cardiomyopathy, gastrointestinal pathology, CNS abnormalities, and death [10]. In the USA, *T. cruzi* infection can be treated with two drugs, benznidazole and nifurtimox, which are exclusively controlled and distributed by the Centers for Disease Control and Prevention (CDC). Concerns with blood-borne transmission of *T. cruzi *led to screening of 65% of the US blood supply for anti-*T. cruzi* antibodies. From 2007 to 2010, this screening resulted in recognition of 1,113 cases of *T. cruzi *infection. Despite recommendation to seek medical treatment, only about 11% of these new *T. cruzi* seropositive blood donors or their physicians have contacted CDC for treatment consultation [1]. The low percentage of treatment consultations indicates a need to understand why so few seropositive donors have sought treatment. One hypothesis may be that these seropositive donors are marginalized Latin American immigrants who have difficulty accessing healthcare.

Testing and treatment for Chagas disease may be difficult among Latinos in the US because they are a marginalized, underserved population who are less likely to seek healthcare [11]. Hispanics are more likely to be poor and uninsured than any other race/ethnicity [12]. As a group, they are less likely than other races to favorably rate their quality of treatment or receive appropriate and timely treatment [11]. The reasons for these disparities include the cost of treatment, legal status, lack of insurance, and lack of interpreters (reviewed in [13]). In addition to the aforementioned structural barriers, many beliefs that are prevalent in the Latino community affect their access to healthcare. Often, Latinos fear the cost of health care, long waiting times, potential for deportation, and discrimination that they associate with accessing healthcare [11]. The combination of structural barriers and beliefs causes Latinos in the USA to use a variety of alternatives to mainstream healthcare, including self-medication and having friends or family members send medicines from their home country in order to avoid seeing a doctor in the USA [11]. 

In summary, due to the rapid growth of the Latino population [14] and their low levels of use of mainstream healthcare, the risk and burden of Chagas disease in the USA are increasing. The goal of this study is to understand the awareness of Chagas disease and identify common healthcare-seeking behaviors and barriers to prevention and treatment of Chagas disease among Latino immigrants in Georgia in order to create effective interventions to prevent and treat Chagas disease. The state of Georgia was chosen as the study area because the Latino population of Georgia is the third fastest growing population in any state, making it the tenth largest Latino population in the USA (8.8% of the state's population) [12, 14] roughly half being foreign born [1]. Focus group discussions with Latino immigrants in Georgia were analyzed to explore when and how Georgia Latinos used mainstream healthcare and their awareness and perceptions of Chagas disease.

## 2. Methodology 

This study was conducted in both urban and rural areas of Georgia. The urban study site was Atlanta, which is one of the largest urban centers in the southeastern US and has a high concentration of Latino residents (approximately 8%), approximately half of whom are foreign born [12]. The rural study site comprised two farms in Moultrie, Georgia, an agricultural area relying heavily on manual farm labor where approximately 13% of the population is Latino [14]. Study participants were male and female immigrants, 18 years or older, who were born and lived in Latin America for at least ten years prior to the study, spoke Spanish, and were currently residing in Georgia. 

Women's perspectives on health-seeking strategies for Chagas disease are important because of their traditional role in healthcare decision making and accessing health services. Additionally, women's awareness of Chagas disease may influence the risk of congenital transmission of the disease. Latina women mainly reside in urban areas of Georgia and were therefore only recruited from the city of Atlanta. As female participants generally did not work outside the home, they were purposively recruited through formal and informal community networks as informed by the study's Community Advisory Board (CAB). The CAB consisted of representatives from the Latin American Association and the Hispanic Health Coalition of Georgia. These organizations were identified by the research team as representative of and influential in the Latino community in Georgia. The CAB advised on the cultural sensitivity of all aspects of the study including the discussion guide, participant recruitment, and dissemination of the findings to the Latino community. Focus group discussions explored when and how Latinos access health care and their awareness of Chagas disease. Formal networks included senior health classes and prenatal classes. The informal network was a group of friends who met to exercise together. The leader of each network was informed about the study and invited researchers to attend a regular meeting to describe the study and invite participation. A total of 45 female participants were recruited for six focus group discussions. 

Men's perspectives about health seeking for Chagas disease are equally important given their limited access to health services. The lack of health insurance and low wages common among Latino men may also influence health-seeking strategies and the ability to pay for healthcare. Latino men were recruited from both urban and rural areas; they typically work in urban areas as day laborers in the informal job market or as unskilled labor in low-wage jobs and in rural areas in the agricultural sector (i.e., migrant farm workers). Day laborers often seek work in public locations; therefore, venue-based recruitment was used in locations where men congregated looking for work (e.g., close to hardware stores, specific gas stations). The study was explained, and eligible, immigrants were invited to participate. Only one focus group with six-day laborers was held due to their priority to continue touting for work. In order to include urban male immigrants who were not day laborers, one focus group was held with eight male immigrants recruited from a church. In the rural study site, men (migrant farm workers) were recruited at a mobile health clinic providing free, on-site care after working hours to immigrants on two farms. Men were approached (after their health care visit) and told about the study and invited to participate. Twenty-three male farmworkers were recruited for three focus group discussions. A total of 37 male participants were recruited for five focus group discussions.

Focus group discussions helped to identify a range of perceptions and experiences on participants' health seeking behaviors. Discussion groups were stratified by gender (five male groups, six female groups) for homogeneity so that participants felt comfortable sharing their perspectives and to allow for analytic comparisons by gender. Data were collected in two rounds: first from May to August of 2011 and second in February of 2012 until saturation of information was reached [15]. All focus group discussions were conducted in Spanish by Spanish-speaking moderators, gender matched to participants, and trained in human subjects research. A note taker was also present at each group discussion. Discussions lasted 60–75 minutes including the completion of the demographic survey. All group discussions were digitally recorded. Focus groups in the urban study site were held in private homes, churches, restaurants, and nongovernmental organization offices. In rural areas, group discussions were held outdoors, under a tent adjacent to the mobile health clinic. In the first round of data collection, participants were given refreshments as compensation for their time; during the second round of collection participants were also compensated with a travel stipend. 

A semistructured discussion guide was developed, translated into Spanish, back-translated, pilot-tested, and refined with the help of the study's CAB before data collection. Additional demographic data were collected from each participant via a brief survey. The survey was also translated into Spanish, pilot-tested, and refined. The survey collected data from each participant on their country of origin, time spent in Latin America, education, and income. Study staff administered the surveys at the end of each group discussion in oral and written forms depending on participants' literacy levels. 

This study was approved by the Emory University Institutional Review Board (IRB 00018964). Oral informed consent was sought from each participant in Spanish. To ensure clarity, participants were asked to summarize their understanding of what participation entailed prior to the discussion. After the discussion, each participant was given a written copy of the oral consent in Spanish. 

All focus group discussions were transcribed verbatim in Spanish, checked for accuracy, deidentified, and uploaded into MAXQDA2007 (Marburg, Germany). The grounded theory approach was used to collect and analyze data, following a cyclical process in order to confirm findings and gain a richer understanding of context of issues that arose [16]. Data were read and annotated to identify core themes from which inductive and deductive codes were developed. All data were then coded by the research team. Intercoder reliability was assessed using an independent researcher who recoded a selection of text with the research team's codebook. Coding discrepancies were identified and addressed. Data were searched by topical themes, and a description encompassing the context, depth, and breadth of core themes in the data was developed. Themes were compared by gender and by urban and rural residence for males to identify differences in health seeking behavior. Four types of health-seeking behaviors were identified: using traditional remedies, delaying health seeking, using mainstream health services, and using alternative health services (e.g., herbalists, physicians in their home country). A conceptual framework of the pathways between these strategies was developed and validated with the data. Core differences in health-seeking pathways were identified between men and women. 

## 3. Results

Focus group discussions were held to understand the awareness of Chagas disease and identify common healthcare-seeking behaviors and barriers to prevention and treatment of Chagas disease among Latino immigrants in Georgia. To understand why Latino immigrants do not seek testing and treatment for Chagas disease, we analyzed data describing when Latino immigrants seek healthcare, their process of seeking healthcare, including the types of behaviors used in seeking care, the motivation for choosing those behaviors, and the pathway between behaviors. Participants were unaware of Chagas disease; thus the moderator asked how participants would seek care if they had symptoms of Chagas disease (e.g., severe pain in their chest or stomach). Participants had not experienced these symptoms; therefore, they could not describe their actual health-seeking strategy; instead, participants described hypothetically how they would seek heath care if these symptoms arose. For example, they described their experience in seeking care for a range of health issues, from minor conditions like colds to severe and chronic conditions like breast cancer. 

Approximately two-thirds of participants (66%) were from Mexico (Table 1), and participants had a median age of 40.5 years (range 19 to 80). Almost half (49%) reported a household income of less than $12,000 per annum (*n* = 29), although there was a high rate of nonresponse for this question (28%). 25% reported their highest level of education completed as primary school or less (Table 1).

### 3.1. Awareness of Chagas Disease

Before describing the process of seeking healthcare by Georgia Latino immigrants, it is important to describe the participants' perceptions of Chagas disease as it affects their motivation to be tested or treated for Chagas disease. All participants, but one, were unaware of Chagas disease. The only participant who had heard of Chagas disease learned of it through her daughter who had a Ph.D. in microbiology. All other participants were unaware of Chagas disease, but the majority were able to report basic knowledge of other vector-borne diseases (e.g., dengue fever and malaria).

When shown photos or a specimen of the triatomine, the vector of Chagas disease, recognition by its commonly known name “*chinche” *was more frequent among male participants. However, the local term “*chinche*” is used to refer to many different insects across Latin America that resembles the triatomine and does not necessarily refer specifically to the Chagas disease vector. Participants debated the name, origin, environment, ecology, and health effects of the bug that they referred to as *“chinche” *suggesting that few participants had specific knowledge of the vector. Both men and women commonly confused the triatomine with bed bugs and attributed a range of health problems to them such as diarrhea and rashes, which are not associated with triatomines. 

### 3.2. Process of Seeking Healthcare

Due to the minimal awareness of Chagas disease amongst participants, the study team sought to identify common healthcare-seeking behaviors and barriers to prevention and treatment of Chagas disease by understanding the process of seeking healthcare for a range of health issues, by Latino immigrants. 

**Figure 1:**
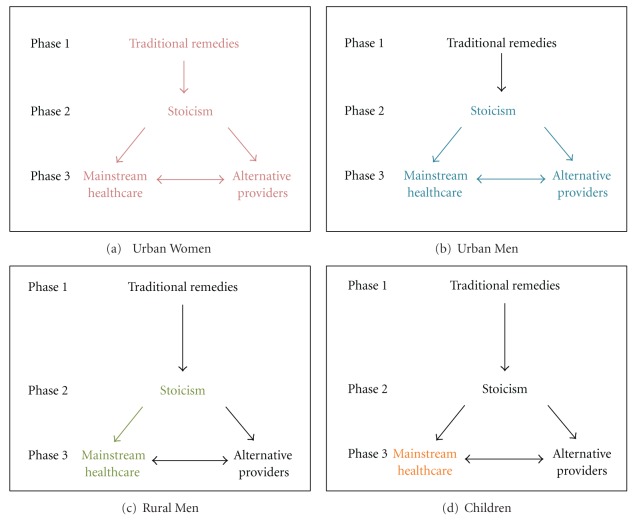
Process of seeking healthcare among focus group participants (Latino immigrants). “Phase” represents the strategy summarizing the healthcare-seeking behavior expressed by the participants. Arrows represent the change in strategy. The process applicable (colored font and arrow) and not applicable (gray font and arrow) to each population of participants is represented. (a) urban women, (b) urban men, (c) rural men, and (d) children.

Participants descriptions of the process of seeking healthcare for a range of health issues, from minor conditions like colds to severe and chronic conditions like breast cancer, highlighted three distinct stages: phase 1 involved the use of traditional remedies, phase 2 involved a period of stoicism, whereby no healthcare was sought, and phase 3 involved consulting a health provider, either a mainstream (i.e., licensed clinics, hospitals, and medical providers) or alternative provider (Figure 1). The pathway through these stages differed by gender and location of residence (rural men differed from urban men). Each stage is described in the following for each type of participant. 

#### 3.2.1. Women

Female participants indicated that their first response to illness was to use traditional remedies (Phase 1, Figure 1(a)), when confronted with familiar and less severe health problems such as colds or stomach aches. They described a variety of traditional remedies. Some were widely perceived as safe and effective, such as brewing various types of tea (e.g., lemon and cinnamon), using herbal compresses, or making remedies out of insects. Participants found other traditional practices used by Latinos to be outlandish including the use of tarot cards to treat heart disease and necklaces of tomatoes to treat sore throats. In general, traditional remedies were used exclusively as the first recourse for illness and were not used in conjunction with over-the-counter medicine from pharmacies. The following quote illustrates the common use of traditional remedies.

* For the most part, we treat ourselves with natural or homemade medicines, but if it doesn't cure us, one goes to the hospital, to the doctor, but first we treat with what we know. (Female, Group 4, P1)*

Women recognized that traditional remedies were not always effective and caused them to delay seeking formal care but continued to use them as the first recourse for illness. Women described using traditional remedies, despite their risk, because it was their cultural tradition and their lower cost compared to using mainstream health providers. Family and friends who were still in Latin America were considered more knowledgeable regarding traditional remedies due to the long history of their use in Latin America. Therefore, women sought advice from family members in their home countries on how to use traditional remedies and had remedies unavailable in the USA mailed to them. The following extract demonstrates how female participants valued the advice of family members in their home countries about traditional remedies.

*Because they [family in country of origin] almost always cure themselves with natural medicines like an herb or something. They make them into tea. They know more, a little more than one [here]. (Female, Group 4, P2)*

In addition to the custom of using traditional remedies, women also preferred to use them because of the lower cost compared to mainstream healthcare providers. Women knew that if the traditional remedies cured their maladies, they saved money, but if they failed it was both dangerous and often more expensive. The following quote demonstrates their preference for traditional remedies due to their lower cost.

*You call [your family] on the phone and you say to them, “my stomach hurts, what can I take? [They say] take this, take this.” If it works you save [the money for] the doctor, but if it doesn't work, that's the bad part. It doesn't save you [money]. (Female, Group 5, P6)*

If traditional remedies were ineffective or unavailable, women described a second phase of health treatment as “*aguantando*” (waiting stoically) (Phase 2, Figure 1(a)), which involved postponing caring for their illnesses, with the hope that the illness would cure itself. Women described four reasons for not seeking healthcare and waiting stoically. First, the lack of economic resources to seek medical care led women to wait for the symptoms to disappear on their own. For example,

*P7: “We wait. When the pain is strong and we can't [wait] anymore, then we [go to the doctor].” Moderator: “Why do you wait?” P8: “Because of the same [reason], because there is no money.” (Female, Group 5, P7-P8)*

Second, women reported the fear of being diagnosed with a fatal or expensive illness as reason for waiting to seek healthcare. Women described how some Latinos prefer not to know if they were ill, even though they suffer and could potentially stop the spread of infection, because they believe they will die anyway due to a lack of resources. For example,

* Because many times, we, I have friends that I tell [them] “let's go get a checkup” and they say “no, I won't because if I have some problem, I am scared and prefer not to know…because if I have something severe, I don't have any money and will die sooner, before my time.” (Female, Group 4, P1)*

Third, some women stated that they waited stoically because they lacked trust in mainstream healthcare providers in the USA. These women described how some providers prioritize earning money over caring for patients and cited examples of providers requiring excessive tests, being unwilling to use test results from other clinics, and charging for consultations without providing treatment. The following extract demonstrates these perceptions.

* When you go to the doctor, they say “I have to do an exam for this,” but sometimes you say “this is just to get my money.” Yes, because sometimes you already went to another clinic and they did a blood test, but in the other clinic [the new one] they repeat the same test to get information that you knew from the beginning. When they do it and you ask why, [they say] “I can't prescribe anything if I don't do the exam to know what you have.” (Female, Group 5, P6)*

The fourth reason for women waiting rather than seeking healthcare was that they felt their health was not a priority for them. They generalized Latinos as “*descuidados*” or careless with their health. Women portrayed Latinos as too lazy or irresponsible to seek healthcare, even if they had the resources to do so. When the moderator described Chagas disease and asked why Latinos would not seek treatment if they knew they were infected, one woman responded as follows.

* There are many people who are lazy that have the help and that have the means and say “ay no.” They prefer to sleep or they prefer to do other things. (Female, Group 3, P6)*

If symptoms became insufferable, women entered into the third phase of the process of seeking healthcare (Phase 3, Figure 1(a)), which involved either consulting alternative health providers or mainstream healthcare providers depending on their available resources. Alternative providers included stores that provide prescription medications (e.g., antibiotics) without a prescription, natural doctors/herbalists who had no formal medical licenses, and doctors in their country of origin who would post prescriptions to the USA. Typically, women who did not have the financial resources to pay for mainstream healthcare used these alternative providers. Women also used alternative providers when they knew what medicines they needed or when they had strong relationships with physicians in their home countries. Some women explained

* I buy [prescription medicines] because they are cheaper than [going to a] doctor. Let's say, for example that you have a sore throat, an infection, that won't go away with pharmacy cough syrup. It won't go away with pills because the infection is very strong. What do we do? We find antibiotics in the Latin stores. We find an injection of penicillin and we inject ourselves with penicillin, amoxicillin, pills. And with this, two shots of penicillin it's over. Sixty dollars and we will cure ourselves. (Female, Group 5, P8)*

* I suffered a lot from my ears… and he [a doctor] charged me about $200…and from then on when we had a problem, we had a doctor in Guatemala where I used to go and I would call him and tell him “I feel like this and he would tell me what to do and my family would try to send it [medicine] to me.” (Female, Group 4, P3) *

Some women chose to use mainstream healthcare rather than alternative providers when waiting became intolerable (Phase 3, Figure 1(a)). Women primarily chose to use mainstream providers if they had sufficient financial resources to pay for healthcare, or if they had very grave illnesses. Yet, within the category of mainstream healthcare, various factors were used to select providers, the most important, across all focus groups, was cost. For those eligible, acceptance of Medicare/Medicaid was the first requirement when searching for doctors. Those who were ineligible for government aid asked for recommendations from acquaintances for the most economic option among mainstream providers. Other women selected providers who offered payment plans and sliding payment scales. One woman described how she selected her provider based on affordability

* It depends on your situation, the situation, what you feel, the symptoms… according to me I had problems in my breast, this breast, and I was thinking, it took me almost two months thinking “where, where, where will it be the cheapest?” (Female, Group 3, P4)*

If mainstream healthcare failed to cure those women who first chose mainstream healthcare over alternative providers (Phase 3, Figure 1(a)), they described turning to alternative providers. Women cited experience (either personal or from acquaintances) where the doctors failed to cure their illnesses either by being too expensive or ineffective. They turned to alternative providers such as “natural doctors” or herbalists or having their doctor from their country of origin send a prescription through the mail (Phase 3, Figure 1(a)). One woman described how her friend turned to alternative providers.

* There is a woman who went to different doctors, even American doctors, and all to see if [her illness] changed but no, [it stayed] the same. Her daughter went to a natural doctor here in Atlanta…she is taking some herbs, some plants that he recommended and is better and can move her leg and arm. (Female, Group 2, P3) *

#### 3.2.2. Children

Women considered the health of their children to be a priority, therefore, when a child was ill, women would directly seek mainstream healthcare, thereby skipping Phases 1 (traditional remedies) and 2 (waiting stoically) and moving straight to Phase 3 (only mainstream providers) in Figure 1(d). One woman cited that even though she knew what was wrong with her child, she would take her child to the doctor.

* Even though [it is just a fever] one goes to the doctor if they have a fever or something and the only thing they [the doctor] tells you is to give them aspirin and it's over. And if you have to pay [no Medicaid] they charge you for the consult and everything. (Female, Group 4, P2) *

Often children had Medicaid so mainstream healthcare was less costly, but women would find the time and the money to take children to mainstream providers, even if they were not eligible for Medicaid. 

#### 3.2.3. Men

In contrast to women, both urban and rural men described their first action when ill to be “*aguantando*” or waiting stoically, rather than attempting to treat their illnesses with traditional remedies. Therefore men entered the process of seeking healthcare at Phase 2 in Figures 1(b) and 1(c) by waiting for the illness to cure itself. Men cited the same reasons as women for waiting rather than seeking healthcare, including a lack of financial resources to pay for healthcare and fear of being diagnosed with a fatal or expensive illness. The following are examples of how Latino men dreaded going to the doctor.

* I think getting sick is a problem for Latinos here in the United States because it costs us a lot. This is the biggest problem there is for [Latinos]. If you get gravely ill, they [doctors] won't see you because it is so expensive for [Latinos]. It [healthcare] is mostly for people from the United States. (Male, Group Rural Men 1)*

* We [Latino men] are much more careless with ourselves. Going to the doctor does not matter to us, we dread going to the doctor, we are always afraid of that they will tell us that we have this thing or the other. (Male, Group Urban Men, P4)*

Men, similar to women, criticized Latinos for being irresponsible with their health, but the men attributed this negligence to a culture of machismo, in which men felt that they should not acknowledge illness in order to maintain their masculinity. Participants described how machismo prevented men from seeking healthcare.

* We are careless [with our health]. Why? Because of machismo, how can we acknowledge it [that we are ill]? [Men say] How could it be? The doctor is mistaken.*

When symptoms became intolerable, men entered Phase 3 in the process of seeking healthcare (Figures 1(b) and 1(c)), but Phase 3 varied by the area where the men lived (urban versus rural). Urban men used both mainstream and alternative providers, while rural men only used mainstream providers, as described in the following.

#### 3.2.4. Urban Men

When waiting became intolerable, urban male participants either sought care with mainstream healthcare providers (e.g., hospital emergency rooms and clinics) or with alternative providers (phase 3, Figure 1(b)). Despite being more expensive, mainstream healthcare was preferred over alternative providers and those who had sufficient funds would seek mainstream healthcare. 

Urban men who did not have the financial resources to pay for mainstream healthcare used alternative providers (Phase 3, Figure 1(b)). These included Latino or Asian stores selling prescription drugs without prescriptions and unlicensed dentists and doctors in Georgia who would treat them at a lower cost. Although urban men recognized the risks of using these alternative providers and expressed concern over their effectiveness and safety, affordability was the priority. If alternative health providers failed to cure them, urban men described using mainstream healthcare providers. The following demonstrates their lack of confidence and their acknowledgment in the risk of delaying care.

* The people will tell you anything. They are not prescriptions of doctors; rather they are prescriptions of the people. You go to the pharmacy, Chinese or Hispanic; in the Hispanic stores around here they will give you a “mejoral,” an aspirin, all that, and if it doesn't work you have to go to the doctor, but your condition is already serious. (Male, Group Urban Men, P2)*

There were two differences in Phase 3 between urban men (Figure 1(b)) and women (Figure 1(a)). First, the type of alternative providers used by those urban men who could not afford mainstream healthcare differed from those used by women. Women described seeking care from physicians in their home countries while urban men described using unlicensed providers in Georgia. Second, unlike women, after mainstream providers failed to cure them, no urban men described turning to alternative providers; they only sought care with other mainstream providers.

#### 3.2.5. Rural Men

Rural men (i.e., migrant farm workers), similarly to urban men, began the process of seeking healthcare directly in Phase 2 of Figure 1(c) by waiting stoically. Yet their reason for waiting was a reluctance to take time off from work rather than fear, dread, carelessness, or lack of funds to pay for care. Rural male participants came to the USA to earn money and were hired on a temporary basis. They were unwilling to seek healthcare unless it was absolutely urgent because taking time off from work meant lost earnings. They preferred to wait and then seek care when they returned to Mexico in order to avoid losing time at work and to ensure continuity of care. When asked why he did not go to a clinic when ill, one participant responded as follows.

* In order not to lose a day [of work], to maintain a relationship with the boss, you have to work. Economically, economically you have to work. This is what we know. You have to take care of yourself, that's how it is. (Male, Group Rural Men 1)*

When waiting became intolerable, rural men entered Phase 3 of Figure 1(c) but only sought care with mainstream health providers. Rural men made no mention of using any type of alternative providers. When selecting mainstream providers, rural men described their choice was based on convenience, in order to avoid losing more time at work. 

### 3.3. Potential Healthcare Seeking for Chagas Disease

#### 3.3.1. Perceptions of Chagas Disease

In order to understand how the process of seeking healthcare would change if the participants were infected with Chagas disease, the study team explained the symptoms and transmission of Chagas disease. After the explanation of Chagas disease, participants expressed concern over being unknowingly infected. This concern stemmed from learning about the lack of definitive symptoms and the long asymptomatic period associated with Chagas disease. The lack of definitive symptoms, the length of the asymptomatic period, and the potential gravity of the disease were identified by participants to be the most important points to communicate regarding Chagas disease because of the difficulties in ascertaining whether or not testing was needed. 

Women expressed more interest in all aspects of Chagas disease compared to men. Only women expressed concern about the possibility and mechanisms of congenital transmission. For example when asked about what they would do if they found they were infected with Chagas disease, one woman responded as follows.

* I am more worried about my daughter because you say that it can be transmitted to children [during pregnancy] as well. (Female, Group 4, P1)*

In addition to women's apprehension regarding vertical transmission, participants highlighted the same barriers in seeking testing and treatment for Chagas disease as seeking care for other health problems. Economic limitations were particularly important. When asked what they would do if they were infected with Chagas disease women responded as follows.

* I would look for medical help.*

* Depending on the cost.*

*Yes because if it costs $22,000 let us say, something really expensive, I will never be able to pay. (Females, Group 3, P5 and P6)*

Another woman emphasized the cost barrier to testing and treatment for Chagas disease when asked why Latinos wouldnot seek testing.

* “The price. Right? It's not that one does not want to go to the doctor… It's because Hispanics generally don't have very much income, and even less than what we have [is available] because generally only the husbands work and everything has to come out of [their salary].” (Female, Group 2, P2)*

In order to identify whether the length or side effects of treatment would be a barrier, the study team explained that the medications for treating Chagas disease had to be taken for three months and often had side effects including tingling, numbness, and rashes. Participants stated that this would not affect their decision to treat their disease, even likening it to the use of contraceptives.

* We know that we take care of ourselves with contraceptives and it has consequences anyways we still take them despite the consequences.*

*Because if we don't then there are other consequences. (Females, Group 4, P1 and P2)*

#### 3.3.2. Modifications to the Process of Seeking Care

As previously mentioned, participants described their process of seeking healthcare for a range of issues because they had no experience seeking care for symptoms similar to those of Chagas disease (severe chest or stomach pain). When asked how this process would differ if they did have symptoms similar to Chagas disease, there was only one difference. Women said that if they and their family or friends were unaware of remedies for severe chest pain then they would skip Phase 1, Figure 1(a), and move directly into stoicism (Phase 2, Figure 1(a)). Additionally, both women and men said that they would be stoic as long as possible, but if the pain was intolerable they would move into Phase 3. 

While participants criticized Latinos for being careless with their health, they excluded themselves from these reproaches by claiming that they prioritized their own health. When asked what they would do if they found out they were infected with Chagas disease, most said that they would find a way to cure it because they valued their health. The following are responses of participants when asked what they would do if they were infected with Chagas disease.

* [I would take] the treatment [for Chagas disease]. I know it is expensive, but I would find the money, if I didn't have it to do the treatment. Because to leave it [untreated] is like not loving yourself. Because if you love yourself and you love your body, logically you will do what's right for it, right? But, many say, “no, it will go away, it will go away” but it doesn't. (Male, Group Urban Men, P1) (Medications to treat Chagas disease are provided at no cost in the United States, but at the time of this quote the group had not been informed of that, and the participant may have assumed that the medication would be expensive or may have been referring to other costs associated with missing work, paying for medical consults, etc.)*

## 4. Discussion

The goal of this study was to understand awareness of Chagas disease and identify common healthcare-seeking behaviors and barriers to prevention and treatment of Chagas disease among Latino immigrants in Georgia from which to develop effective prevention and treatment interventions. Four barriers to being tested and treated for Chagas disease were reported by study participants including (1) a lack of awareness of the disease signifying a lack of motivation to be tested, (2) fear of being diagnosed with a fatal disease or an illness requiring expensive treatment, (3) reservations about the quality of care from mainstream healthcare providers among women and urban men, and (4) economic limitations to accessing mainstream healthcare. Finally, women described seeking mainstream healthcare immediately if their children became ill; thus no barriers were found to treating Chagas disease among children.

### 4.1. Lack of Awareness Regarding Chagas Disease

One of the prominent findings of this study was that the lack of awareness of Chagas disease was a significant barrier to testing. Currently, the authors are unaware of any studies measuring awareness of Chagas disease among Latino immigrants in the USA. However, a qualitative study in Spain among at-risk immigrants also demonstrated a lack of knowledge, fears, and false beliefs about Chagas disease [17]. The lack of awareness exhibited by participants in this study may be due to the low prevalence of Chagas disease in the USA, as few immigrants have been diagnosed with Chagas disease (previous studies have documented seroprevalence rates ranging from 0.4% to 4.9%) [6–8]. Additionally, awareness of Chagas disease is limited among medical providers in the USA; 68.2% of American College of Obstetricians and Gynecologists surveyed described their knowledge level about Chagas disease as “very limited” [18]. Yet, in areas where the disease is endemic, such as Argentina, there have been high rates of public participation in vector control campaigns which indicate an awareness of risk of Chagas disease [19]. 

The lack of awareness of Chagas disease documented in this study suggests a need for increased education about risk factors for contracting the disease and the need for testing due to a lack of definitive symptoms and an extended asymptomatic period. In the USA, there is only one educational campaign aimed at educating Latino immigrants in California about Chagas disease, in which health promoters educate them about their risk and encourage testing [20]. In Spain, the number of consultations for Chagas disease doubled after instituting a community-based educational program using leaflets. Finally new efforts have been made in the USA; the CDC has created a training module to educate medical providers and brochures printed in Spanish to inform Latin American immigrants about Chagas disease [21]. Participants in our study and others described their fears of being diagnosed with a fatal disease or illness that is expensive to treat [22, 23]. Increased education concerning the availability of free treatment may allay these fears and may also encourage testing. 

### 4.2. Economic Limitations

In addition, our study, similar to others [11, 24], highlighted that mainstream healthcare was not sought because of the high cost and lack of health insurance. Among urban Latinos, our study and others found that the use of alternative providers (e.g., folk healers, homeopathic practitioners, and nonphysician prescribed medications) to overcome these structural barriers associated with mainstream care is common [11, 22, 25]. Increasing low cost and free services as well as other forms of support such as medical insurance for immigrants has been shown to be an effective way to increase access to healthcare among uninsured Latino immigrants [26]. Among migrant farm workers, our study and others [27] found that the primary structural barrier for seeking care was not cost as described by urban participants, but losing time at work and subsequent earnings. In rural areas, offering screenings, education, and preventive care in mobile clinics after work hours has been shown to increase access to mainstream healthcare for rural male immigrants [28–30]. 

### 4.3. Quality of Care

A further barrier to testing and treatment seeking for Chagas disease was reservation about the quality of care they would receive from mainstream healthcare providers. Often, Latinos fear the cost, potential for deportation, long waiting time, and discrimination that they associate with accessing healthcare [11] and consistently rate their experiences with medical staff more poorly than non-Hispanic whites [31]. This indicates a need for educating mainstream providers about ways to increase cultural sensitivity within their practices. Some educational resources for healthcare providers are already available and have been shown to increase overall knowledge and confidence in Latino cultural beliefs related to healthcare [32]. 

### 4.4. Use of Alternative Providers

Due to their inability to pay for care, urban participants described using a variety of alternative providers due to the lower cost associated with these providers. Some of these alternative providers practiced forms of complementary and alternative medicines (homeopathic healers and herbalists) which are increasingly being incorporated into mainstream healthcare [33–35]. Existing educational modules for improving cultural sensitivity amongst health providers incorporate awareness of alternative medicines. However, forming partnerships with some of the alternative providers identified (e.g., stores that sell prescription medications without prescriptions and physicians without licenses) is not feasible due to the unsafe and illegal nature of their practices. 

No rural men described using alternative providers in order to bypass the barriers of seeking mainstream care. All the rural male participants were in Georgia on H-2A visas, which are temporary visas in which farmers hire, transport and house immigrants during the agricultural growing season, after which they are returned to their home countries [36]. Given the temporary and restricted nature of the farm workers' time in Georgia, it is possible that their lack of transportation may have limited their access to alternative providers. Additionally, farm workers might not have been aware of alternative providers, because of their limited time in Georgia and the isolated nature of being housed on farms. The dependence of rural men on mainstream healthcare providers when they are ill is an opportunity to increase preventive care for this population.

Finally, we found that mothers reported immediately taking their ill children to mainstream healthcare providers regardless of cost or other barriers. This practice is in contrast to previous studies which show that very few Latina mothers seek care for their children in a timely and appropriate manner [37, 38]. This process of immediately seeking mainstream healthcare for children may be a new opportunity to increase access to healthcare for women and their families. Although the authors are currently unaware of any targeted efforts to integrate care for Latina mothers and their children in the USA, integration of medical services has been shown to be effective in diverse settings and for a variety of preventive practices [39–42]. 

### 4.5. Potential Healthcare Seeking for Chagas Disease

A conceptual framework (Figure 1) was developed to understand the process of seeking healthcare by Latino immigrants and to understand the context of the barriers to testing and treatment for Chagas disease that were identified by participants. Some of the types of behaviors identified in this framework have been previously documented [11, 22, 24, 25], but the authors are unaware of any existing frameworks describing the pathways between behaviors and the applicable contexts of seeking healthcare among Latino immigrants. While participants were unable to describe their process of seeking healthcare specifically for Chagas disease, the model was consistent across groups and for all health problems mentioned by participants; thus, this model should also be applicable to Chagas disease.

Latino immigrants described seeking mainstream healthcare only when they could no longer tolerate symptoms of illnesses. Therefore, the lack of definitive symptoms and long asymptomatic period associated with Chagas disease means that Latino immigrants would not seek care for Chagas disease until they enter the clinically evident stage of the disease (e.g., cardiomyopathy, achalasia, and megacolon). Based on this model, upon entering the clinically evident stage of Chagas disease, a female Latina immigrant in Georgia would attempt to treat herself with traditional medicine. When traditional remedies failed, she would wait until the symptoms were unbearable and then seek care from either mainstream providers or alternative providers depending on her financial resources. Urban and rural male Latino immigrants would not use traditional remedies to treat themselves; rather they would wait until symptoms became unbearable and then seek care. Urban males would seek care with either mainstream or alternative providers depending on their financial resources, but rural males would only seek care from their closest mainstream provider. Finally, if a Latino child exhibited symptoms of Chagas disease, women would immediately take them to a mainstream provider. 

#### 4.5.1. Strengths and Limitations

The qualitative approach and inclusion of difficult to reach populations (e.g., Latino day laborers and migrant farm workers) in this study allowed a more comprehensive understanding of the perceptions of Chagas disease and the process of seeking healthcare among Latino immigrants. The lack of awareness of Chagas disease and experience of symptoms similar to that of Chagas disease (e.g., severe chest or stomach pains) among participants limited their ability to describe specific healthcare seeking for Chagas disease. Future research should explore how the gravity and familiarity with symptoms of any illness affect the process of seeking healthcare in order to confirm the applicability of the conceptual framework for different types of illnesses. 

## 5. Conclusion

This study provides an in-depth understanding of the barriers regarding testing and treatment for Chagas disease among Latino immigrants by describing their processes of seeking healthcare, the motivations for using these processes, and the pathways between these processes. Latino immigrants tend to wait until they are gravely ill before seeking care due to beliefs and economic limitations. Financial resources primarily determine the process that Latinos use to seek health care. Educational campaigns on Chagas disease need to be implemented based on these treatment seeking pathways (e.g., combining education with Latino children medical visits) in order to increase testing and treatment. In addition, more low cost and free services or health insurance need to be made available for Latino immigrants in order to increase access to preventive care. 

## Figures and Tables

**Table 1:** Demographic characteristics of focus group participants.

	% (*n*)
Country of origin (*n* = 74)	
Mexico	66% (49)
Central America	20% (15)
South America	14% (10)
Education (*n* = 72)	
Primary	25% (18)
Secondary	39% (28)
University	36% (26)
Yearly income (*n* = 59)	
<$12,000	49% (29)
$12,000–$24,000	37% (22)
>$24,000	14% (8)
